# Systematics and Taxonomy of *Tonatia saurophila* Koopman & Williams, 1951 (Chiroptera, Phyllostomidae)

**DOI:** 10.3897/zookeys.915.46995

**Published:** 2020-02-24

**Authors:** Mateo Basantes, Nicolás Tinoco, Paúl M. Velazco, Melinda J. Hofmann, Miguel E. Rodríguez-Posada, M. Alejandra Camacho

**Affiliations:** 1 Sección de Mastozoología, Museo de Zoología, Facultad de Ciencias Exactas y Naturales, Pontificia Universidad Católica del Ecuador, Quito, Pichincha, Ecuador Pontificia Universidad Católica del Ecuador Quito Ecuador; 2 Department of Mammalogy, American Museum of Natural History, Central Park West at 79th St., New York, NY 10024, USA American Museum of Natural History New York United States of America; 3 Department of Biology, Arcadia University, 450 S. Easton Rd., Glenside, PA 19038, USA Arcadia University Glenside United States of America; 4 Fundación Reserva Natural La Palmita, Centro de Investigación, Grupo de Investigaciones Territoriales para el Uso y Conservación de la Biodiversidad, Bogotá, Colombia Grupo de Investigaciones Territoriales para el Uso y Conservación de la Biodiversidad Bogotá Colombia

**Keywords:** Andes, Phyllostominae, *Tonatia saurophila*, *T. saurophila bakeri*, *T. saurophila maresi*

## Abstract

The Stripe-headed Round-eared bat, *Tonatia saurophila*, includes three subspecies: *Tonatia saurophila saurophila* (known only from subfossil records in Jamaica), *Tonatia saurophila bakeri* (distributed from southeastern Mexico to northern Colombia, Venezuela west and north of the Cordillera de Mérida, and northwestern Ecuador), and *Tonatia saurophila maresi* (distributed in Venezuela east and south of the Cordillera de Mérida, the Guianas, Trinidad and Tobago, northeastern Brazil, and along the upper Amazon basin in Colombia, Ecuador, Peru, and Bolivia). The last two subspecies are an attractive example to test predictions about the historical role of the Andes in mammalian diversification. Based on morphological descriptions, morphometric analyses, and phylogenetic reconstruction using the mitochondrial gene Cyt-*b* and the nuclear exon RAG2, this study evaluates the intraspecific relationships within *Tonatia saurophila* and the taxonomic status of the taxon. The three subspecies of *T. saurophila* are recognizable as full species: *Tonatia bakeri*, *Tonatia maresi*, and *Tonatia saurophila*. The latter is restricted to its type locality and possibly is extinct. *Tonatia bakeri*, in addition to being larger than *T. maresi*, is morphologically distinguishable by possessing an acute apex at the posterior edge of the skull, a well-developed clinoid process, and relatively robust mandibular condyles, and by lacking a diastema between the canine and the first lower premolar. The genetic distance between *T. bakeri* and *T. maresi* is 7.65%.

## Abstract (es)

El Murciélago de orejas redondas de cabeza rayada, *Tonatia saurophila*, incluye tres subespecies: *Tonatia saurophila saurophila* (conocida sólo por registros subfósiles en Jamaica), *Tonatia saurophila bakeri* (distribuida desde el sureste de México hasta el norte de Colombia, Venezuela al oeste y norte de la Cordillera de Mérida, y el noroeste de Ecuador), y *Tonatia saurophila maresi* (distribuida en Venezuela al este y sur de la Cordillera de Mérida, las Guayanas, Trinidad y Tobago, el noreste de Brasil, y la vertiente amazónica de los Andes de Colombia, Ecuador, Peru y Bolivia). Las dos últimas subespecies representan un ejemplo atractivo para poner a prueba predicciones sobre el rol histórico de los Andes en la diversificación de mamíferos. Con base en descripciones morfológicas, análisis morfométricos y una reconstrucción filogenética empleando el gen mitocondrial Cyt-*b* y el gen nuclear RAG2, este estudio evalúa las relaciones intraspecíficas dentro de *Tonatia saurophila* y el estatus taxonómico del taxón. Las tres subespecies de *T. saurophila* son reconocidas como especies plenas: *Tonatia bakeri*, *T. maresi* y *T. saurophila*. Esta última está restringida a la localidad tipo y posiblemente está extinta. *Tonatia bakeri*, además de ser de mayor tamaño que *T. maresi*, se diferencia morfológicamente por poseer un ápice agudo en el borde posterior del cráneo, un proceso clinoideo bien desarrollado y cóndilos mandibulares relativamente robustos, y por carecer de un diastema entre el canino y el primer premolar inferior. La distancia genética entre *T. bakeri* y *T. maresi* es 7.65%.

## Introduction

The Neotropical bat genus *Tonatia* Gray, 1827 (Phyllostomidae, Phyllostominae) includes two species: *T. saurophila* and *T. bidens*. This genus is widely distributed from the southern Mexico to northern Argentina and Paraguay (Williams and Genoways 2008). Members of the genus *Tonatia* are easily differentiated from other phyllostomines by a combination of the following characteristics: a single pair of lower incisors; three pairs of lower premolars; a tail that extends to the middle of the uropatagium; short and wide ears that are not joined by a band of skin on the forehead; absence of a notch on the lower margin of the pinna; hairy face, ears, forearms, legs, and feet; and postorbital constriction greater than 5 mm (Williams et al. 1995; Williams and Genoways 2008).

Since its description in 1823 the taxonomic history of *Tonatia* has been controversial. Spix (1823) described *Vampyrus bidens* based on a single specimen from Bahia, Brazil. Four years later, Gray (1827) described the genus *Tonatia* and considered *V. bidens* as its type species. A decade later, Gray (1838) described *Phyllostoma childreni*, a species with similar characteristics to *V. bidens*, based on a single specimen without a precise location in South America. Concurrently, d’Orbigny (1836) described the genus *Lophostoma* based on a specimen of *L. silvicola* collected in Bolivia, the only species he included in the genus (d’Orbigny 1836; d’Orbigny and Gervais 1847). Dobson (1878) listed three species for *Lophostoma*: *L. bidens* (Spix 1823), *L. brasiliense* Peters, 1867, and *L. amblyotis* Peters, 1867; he also listed *L. silvicolum* as a junior synonym of *L. amblyotis*. Additionality, Dobson (1878) synonymized *V. bidens* and *P. childreni* under the name *Lophostoma bidens*. Based on Dobson’s taxonomic arrangement and because *L. bidens* was assigned to the genus *Tonatia* Gray, 1827, Palmer (1898) treated *Lophostoma* as a junior synonym of *Tonatia* Gray, 1827. More than a century later, Koopman and Williams (1951) described *Tonatia saurophila*, based on fragmentary subfossil material found by H. E. Anthony (1919–1920) in two caves in Jamaica (Wallingford and Dairy). Later, Koopman (1976) considered *T. saurophila* as a subspecies of *Tonatia bidens*. The synonymy of *Lophostoma* under *Tonatia* was not challenged until studies of G-banded karyotypes, allozymes, and albumin immunology (Patton and Baker 1978; Baker and Bickham 1980; Arnold et al. 1983; Honeycutt and Sarich 1987) showed that the *T. bidens* complex was divergent from the other species of the genus. Lee et al. (2002) examined DNA sequence variation in the mitochondrial ribosomal genes and found significant differentiation and paraphyly within the genus *Tonatia*. Based on these results, Lee et al. (2002) recommended restricting *Tonatia* to *T. bidens* and *T. saurophila* and transferring *brasiliense*, *carrikeri*, *evotis*, *schulzi*, and *silvicola* to *Lophostoma*. Based on morphological and morphometric differences, Williams et al. (1995) elevated *T. bidens saurophila* from subspecies to species level, resulting in *Tonatia* to include two species: *Tonatia bidens* and *T. saurophila*. Furthermore, Williams et al. (1995) described two subspecies in *T. saurophila*: *T. s. bakeri* and *T. s. maresi*.

The Stripe-headed Round-eared bat, *Tonatia saurophila*, is characterized by presenting a secondary process in the mastoid that partially obscures the base of the mastoid bulla. It also presents a conspicuous space between the cingula of the lower canines, lower premolars relatively uncrowded with the first lower premolar slightly overlapping on the second premolar, and a clear line of short fur on top of the head stretching laterally between the eyes and crown (Williams et al. 1995; Tirira 2017). Currently three subspecies are recognized: *T. saurophila saurophila* (only subfossil records; type locality: Balaclava, Jamaica); *T. saurophila bakeri*, distributed from southern Mexico southward into South America to northern and western Colombia, northwestern Ecuador, and Venezuela (west and north of the Cordillera de Mérida); and *T. saurophila maresi* distributed in Venezuela (east and south of the Cordillera de Mérida), the Guianas, northeastern Brazil, and along upper Amazon basin of Colombia, Ecuador, Peru, Bolivia, and Brazil (Williams et al. 1995; Williams and Genoways 2008).

*Tonatia saurophila* is in need of a taxonomic and systematic revision. Its wide geographic distribution in Central and South America, which includes populations on both sides of the Andes, raises questions regarding the role of this mountain range as a potential barrier to gene flow and a promoter of diversification. The phylogenetic relationships between the species of *Tonatia*, and within the subspecies of *T. saurophila*, have not been investigated. Herein, we aim to evaluate the taxonomic status of the subspecies of *T. saurophila* based on morphological, morphometric, and molecular data. We discuss the role of the Andes in the diversification and the taxonomic substructure of this taxon in light of the results.

## Methods

### Material included

Specimens examined and tissues used in this study are deposited in the following institutions: AMNH, American Museum of Natural History, New York, USA; CEBIO, Centro de Ecología y Biodiversidad, Lima, Peru; FMNH, Field Museum of Natural History, Chicago, USA; ICN, Mammals Collection “Alberto Cadena García” at Instituto de Ciencias Naturales de la Universidad Nacional, Bogotá, Colombia; INABIO, Instituto Nacional de Biodiversidad, Quito, Ecuador; MEPN, Museo de Historia Natural Gustavo Orcés V., Escuela Politécnica Nacional, Quito, Ecuador; MUSM, Museo de Historia Natural, Universidad Nacional Mayor de San Marcos, Lima, Peru; QCAZ, Sección Mastozoología, Museo de Zoología, Pontificia Universidad Católica del Ecuador, Quito, Ecuador.

### Sampling and measurements

We examined 137 adult specimens of *Tonatia saurophila* (68 females, 67 males, and two specimens of undetermined sex), of which 31 were collected in localities west of the Andes, and 106 from localities east of the Andes (Appendix 1). We evaluated 21 craniodental and external measurements based on the external and osteological characteristics defined by Williams et al. (1995) and Velazco and Gardner (2012). The measurements of the body and skull were taken from the left side and of the jaw from the right side. All measurements were made using digital calipers with an accuracy of 0.01 mm. The craniodental and external measurements used in this study were: FA, forearm length; METIII, metacarpal III length; METIV, metacarpal IV length; METV, metacarpal V length; TiL, tibia length; HF, hind-foot length; GLS, greatest length of skull; CB, condylobasal length; CCL, condylocanine length; CIL, condyloincisive length; BB, braincase breadth; PB, postorbital constriction breadth; MPW, mastoid process width; ZB, zygomatic breadth; BC, breadth across upper canines; PL, palatal length; DENL, dentary length; COH, coronoid height; MANDL, mandibular toothrow length; MTRL, maxillary toothrow length; and M2M2, width at M2s.

### Morphological and morphometric analyses

In order to identify morphological differences among *T. saurophila* populations across their range, 23 specimens from localities west of the Andes and 77 from localities east of the Andes were examined. From these observations, each individual was examined and variation patterns of various qualitative and discrete characteristics were described, following Velazco (2005). Descriptive statistics (mean, standard deviation, and minimum and maximum values) were obtained from all the measured specimens. Data transformation was performed through a standardization process. Differences between sexes and between subspecies were evaluated by Principal Component Analysis (PCA) and Discriminant Function Analysis (DFA). Factorial points of these multivariate tests were graphically plotted in morphospace to show relationships between subspecies. All tests were done using the statistical software package PAST v. 1.0 (Hammer et al. 2001).

### Molecular analyses

Tissue samples from 15 specimens stored in the QCAZ mammal collection were used for the molecular analysis. DNA was obtained from liver, muscle, or tail preserved in 95% ethanol and stored at −80 °C, as well as from dried skin fragments from specimens in fluid. The DNA was extracted using the salt protocol (Bilton and Jaarola 1996), modified in the use of 300 μl of ethanol for the washes instead of 1000 μl. The concentration and quality of DNA was measured using the NanoDropTM1000 v. 3.7 spectrophotometer (Thermo Scientific). From the stock solution, aliquots of 20 ng/μl of DNA concentration were prepared to be used in PCR reactions. Sequences of the Cytochrome *b* (Cyt-*b*) and the Recombination Activating Gene (RAG2) were amplified and sequenced for this study. The following primer pairs were used for the Cyt-*b* gene: forward primer glo7L and reverse primer glo6H, and for RAG2: forward primer RAG2-F1 and reverse primer RAG2-R1. Thermal profile for the Cyt-*b* PCR followed Hoffmann and Baker (2001), and for RAG2, PCR followed Baker et al. (2000). The amplicons were visually evaluated with gel electrophoresis and subsequently purified with ExoSap-IT (GE Healthcare, Chalfont St. Giles, UK). Amplicons were then sequenced by Sanger method at Macrogen Inc. (Seoul, South Korea).

Sequences were edited using Geneious R11 (https://www.geneious.com), and aligned using the ClustalW tool. We calculated interspecific and intraspecific genetic distances using software MEGA v. 7.0 (Kumar et al. 2015); to get corrected distances we used the Kimura 2 Parameters. The best partition strategies along with corresponding models of evolution were obtained in PartitionFinder v. 1.1 (Lanfear et al. 2012). For the Bayesian Inference Analysis (BI) the best substitution models for Cyt-*b* were: first position K80+G, second position HKY+I and third position GTR+I. For RAG2 they were: first and second position HKY+G and for third position K80+G, while for the Maximum Likelihood Analysis (ML) substitution model used was GTR. The ML analysis was conducted using RAXML (Stamatakis 2014). Nodal support was determined by 1000 bootstrap replicates. The BI analysis was conducted using MrBayes v. 3.2.2 (Ronquist et al. 2012). Four Markov chains were run twice for 10,000,000 generations. Trees were sampled every 1,000 generations resulting in 20,000 trees saved per analysis. Adequacy of chain mixing was assessed by examining effective sample sizes (ESS) in Tracer, with ESS > 200 considered as satisfactory and plotting the –ln L per generation. After analyzing convergence, chain mixing, and sampling, the first 1000 trees sampled were discarded as “burn-in”. The remaining trees were used to obtain a consensus tree by 50% majority rule. To evaluate the monophyly and phylogenetic relationships of our *Tonatia* samples, several phyllostomines were selected as outgroup (Table 1), which have been used in previous works (Lee et al. 2002; Velazco and Cadenillas 2011; Camacho et al. 2016). The outgroup sequences were obtained from GenBank (www.ncbi.nlm.nih.gov/Genbank).

**Table 1.** Specimens used for phylogenetic analyses. Species, museum and tissue ID numbers, and GenBank accession numbers are given for the *Tonatia* and outgroup samples used in the phylogenetic analyses presented in this study. *Misidentified specimens.

Species	Specimen Catalog #	GenBank #		GenSeq Nomenclature
		Cyt-*b*	RAG2	
*Chrotopterus auritus*	CMNH68638	NA	AF316442	genseq-4 RAG2
	CMNH76767	FJ155481	NA	genseq-4 Cyt-*b*
	AMNH / AMCC110459	KC783057	NA	genseq-4 Cyt-*b*
	FURB-SLA1799	NA	DQ903851	genseq-4 RAG2
*Gardnerycteris crenulatum*	TTU33287	NA	AF316472	genseq-4 RAG2
	MN36684	NA	DQ903850	genseq-4 RAG2
	CMNH25230	FJ155478	NA	genseq-4 Cyt-*b*
*Gardnerycteris keenani*	MUSM19346 /RCO360	MG018960	MG018969	genseq-4 Cyt-*b*, RAG2
	MUSM19347 /SVS0606	MG018961	MG018970	genseq-4 Cyt-*b*, RAG2
	MUSM19190 / ESP003	MG018962	MG018971	genseq-4 Cyt- *b*, RAG2
	MUSM19348 / VPT2959	MG018963	MG018972	genseq-4 Cyt-*b*, RAG2
*Gardnerycteris koepckeae*	MUSM41327 / EA216	MG018959	MG018968	genseq-4 Cyt-*b*, RAG2
*Lophostoma brasiliense*	AMNH267103	NA	AF316489	genseq-4 RAG2
	QCAZ12957	NA	MN585262	genseq-4 RAG2
	QCAZ13837	NA	MN585258	genseq-4 RAG2
	QCAZ15777	NA	MN585254	genseq-4 RAG2
	ROM106608	FJ155486	NA	genseq-4 Cyt-*b*
	NA	JF923842	NA	genseq-4 Cyt- *b*
	AMNH / AMCC110343	NA	KC783118	genseq-4 RAG2
*Lophostoma carrikeri*	ROM107190	JF923843	NA	genseq-4 Cyt- *b*
	ROM107391	JF923844	NA	genseq-4 Cyt- *b*
	QCAZ13578	KU886210	NA	genseq-4 Cyt- *b*
	QCAZ13994	KU886211	NA	genseq-4 Cyt- *b*
	QCAZ4935	KU886212	NA	genseq-4 Cyt- *b*
*Lophostoma evotis*	TTU61070 / TK40341	NA	AF442080	genseq-4 RAG2
	ROM95625	FJ155491	NA	genseq-4 Cyt- *b*
	ROM95626	JF923845	NA	genseq-4 Cyt- *b*
	TTU84384	JF923846	NA	genseq-4 Cyt- *b*
*Lophostoma occidentalis*	MUSM19334	JF923847	NA	genseq-4 Cyt- *b*
	TTU85277	JF923848	NA	genseq-4 Cyt- *b*
	TTU85292 / QCAZ6500	JF923849	NA	genseq-4 Cyt- *b*
*Lophostoma schulzi*	TK18833 / AMNH267106	NA	AF442079	genseq-4 RAG2
	F38318	FJ155485	NA	genseq-4 Cyt- *b*
	ROM101128	JF923850	NA	genseq-4 Cyt- *b*
*Lophostoma silvicolum*	TK56716	FJ155493	AF442081	genseq-4 Cyt- *b*, RAG2
	TK56635	JF923852	AF442082	genseq-4 Cyt- *b*, RAG2
	TK18832 / AMNH267107	NA	AF442083	genseq-4 RAG2
	ROM100949	FJ155492	NA	genseq-4 Cyt- *b*
	MSB68337	JF923851	NA	genseq-4 Cyt- *b*
	CM63684	JF923853	NA	genseq-4 Cyt- *b*
	CM63669	JF923854	NA	genseq-4 Cyt- *b*
	TTU84904	JF923855	NA	genseq-4 Cyt- *b*
	TTU84930	JF923856	NA	genseq-4 Cyt- *b*
	ROM104232	JF923857	NA	genseq-4 Cyt-b
	F38068	JF923858	NA	genseq-4 Cyt-b
	FMNH203542	JF923859	NA	genseq-4 Cyt-b
	CM98608	JF923860	NA	genseq-4 Cyt-b
	CM78340	JF923861	NA	genseq-4 Cyt-b
	CM78337	JF923862	NA	genseq-4 Cyt-b
	AN1918	DQ903830	DQ903849	genseq-4 Cyt-b, RAG2
	T4497	NA	HG380330	genseq-4 RAG2
*Mimon bennetti*	MN36387	DQ903832	NA	genseq-4 Cyt- *b*
*Mimon cozumelae*	ROM96534	NA	KM362064	genseq-4 Cyt- *b*
*Phyllostomus discolor*	NA	HM470153	NA	genseq-4 Cyt- *b*
	NA	HM470154	NA	genseq-4 Cyt- *b*
	NA	HM470155	NA	genseq-4 Cyt- *b*
	NA	HM470156	NA	genseq-4 Cyt- *b*
	NA	HM470157	NA	genseq-4 Cyt- *b*
	ROM112692	NA	KM362066	genseq-4 RAG2
Phyllostomus discolor*	Pdis5655	NA	FN641681	genseq-4 RAG2
*Phyllostomus elongatus*	AMNH / AMCC110396	KC783056	NA	genseq-4 Cyt- *b*
	C2739	KU295471	NA	genseq-4 Cyt- *b*
*Phyllostomus hastatus*	CMNH78333	FJ155479	AF316479	genseq-4 Cyt- *b*, RAG2
*Phylloderma stenops*	CMNH63614	NA	AF316480	genseq-4 RAG2
	QCAZ13589	NA	MN585261	genseq-4 RAG2
*Tonatia bakeri*	QCAZ9233	MN585249	NA	genseq-4 Cyt- *b*
	ROM104215	NA	MN585268	genseq-4 RAG2
	QCAZ9236	NA	MN585270	genseq-4 RAG2
	QCAZ2350	MN585247	MN585274	genseq-4 Cyt- *b*, RAG2
	QCAZ8627	MN585248	MN585272	genseq-4 Cyt- *b*, RAG2
	QCAZ9234	MN585250	MN585271	genseq-4 Cyt- *b*, RAG2
*Tonatia bidens*	TK56633	FJ155489	AF442087	genseq-4 Cyt- *b*, RAG2
	MVZ185673	FJ155490	AF442088	genseq-4 Cyt- *b*, RAG2
	MVZ185959	JF923863	AF442089	genseq-4 Cyt- *b*, RAG2
Tonatia bidens*	MN37301	DQ903829	NA	genseq-4 Cyt- *b*
*Tonatia maresi*	NA	NA	AF203763	genseq-4 RAG2
	ROM103210 / TK49889	NA	AF442084	genseq-4 RAG2
	TK46028	NA	AF442085	genseq-4 RAG2
	TK49885 / NK30034/ MSB70286	NA	AF442086	genseq-4 RAG2
	T4488	NA	HG380332	genseq-4 RAG2
	ROM103210	FJ155488	NA	genseq-4 Cyt- *b*
	ROM103414	JF923864	NA	genseq-4 Cyt- *b*
	ROM119530	MN585236	NA	genseq-4 Cyt- *b*
	ROM125481	MN585237	NA	genseq-4 Cyt- *b*
	ROM125512	MN585238	NA	genseq-4 Cyt- *b*
	QCAZ10950	MN585241	NA	genseq-4 Cyt- *b*
	QCAZ11797	MN585242	NA	genseq-4 Cyt- *b*
	QCAZ12925	MN585243	NA	genseq-4 Cyt- *b*
	QCAZ13364	MN585253	NA	genseq-4 Cyt- *b*
	ROM105914	NA	MN585267	genseq-4 RAG2
	ROM122096	NA	MN585266	genseq-4 RAG2
	QCAZ14405	NA	MN585257	genseq-4 RAG2
	ROM104459	MN585234	MN585269	genseq-4 Cyt- *b*, RAG2
	ROM112583	MN585235	MN585265	genseq-4 Cyt- *b*, RAG2
	ROM125100	MN585239	MN585264	genseq-4 Cyt- *b*, RAG2
	ROM125926	MN585240	MN585263	genseq-4 Cyt- *b*, RAG2
	QCAZ13787	MN585244	MN585260	genseq-4 Cyt- *b*, RAG2
	QCAZ13788	MN585245	MN585259	genseq-4 Cyt- *b*, RAG2
	QCAZ14407	MN585252	MN585256	genseq-4 Cyt- *b*, RAG2
	QCAZ14606	MN585246	MN585255	genseq-4 Cyt- *b*, RAG2
	QCAZ7017	MN585251	MN585273	genseq-4 Cyt- *b*, RAG2
*Trachops cirrhosus*	TK19132	FJ155483	NA	genseq-4 Cyt- *b*
	AMNH267129	DQ233669	AF316490	genseq-4 Cyt- *b*, RAG2
	MN36720	DQ903828	DQ903852	genseq-4 Cyt- *b*, RAG2
	NMNH584479	NA	KF569355	genseq-4 RAG2
*Vampyrum spectrum*	TTU61070 / TK40370	FJ155482	AF316495	genseq-4 Cyt- *b*, RAG2

## Results

### Morphometric analyses

**Figure 1.**
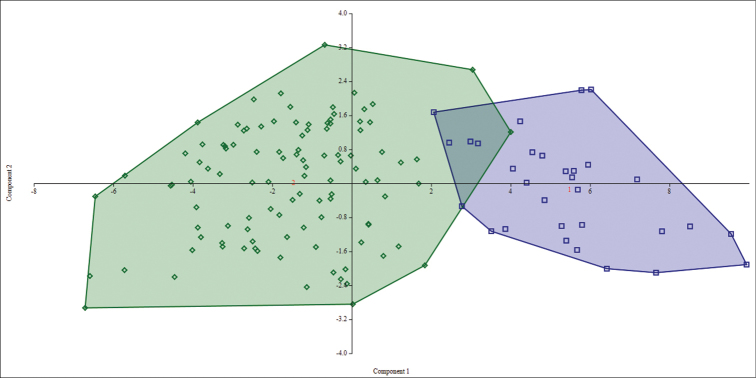
Principal Component Analysis (PCA) of two morphometric groups. Projection of 137 specimens of *Tonatia saurophila* from western (squares) and eastern (rhombuses) localities with respect to the Andes on PC1 and PC2 of a Principal Component Analysis with 21 morphometric cranio-dental and external variables.

**Figure 2.**
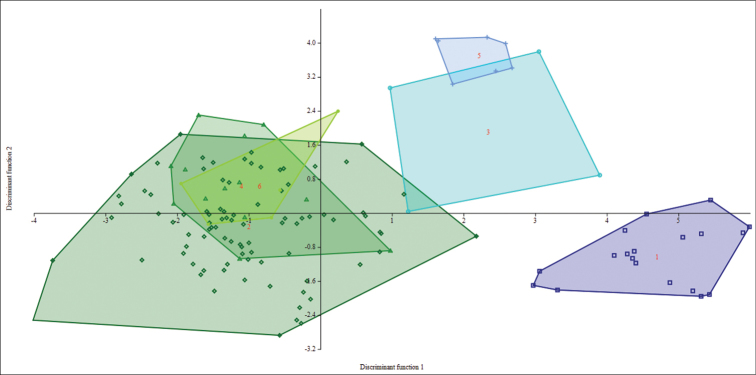
Discriminant Function Analysis of six morphometric groups. Projection of 137 specimens of *Tonatia saurophila*, assembled in six groups according to their localities of origin: western Colombia and Ecuador (squares); eastern Colombia, Ecuador, and Peru (diamonds); Caribbean of Colombia and Venezuela (circles); Guyana, French Guiana, and northeastern Brazil (ovals); Central America (crosses); and Trinidad and Tobago (triangles), on DF1 and DF2 of a Discriminant Function Analysis with 21 morphometric cranio-dental and external variables.

The statistical analyses performed on data obtained from measurements of the entire sample set of *Tonatia saurophila* found no sexual dimorphism within groups for the analyzed variables (contrary to that observed in *Tonatia bidens* and in some species of the genus *Lophostoma*; Davis and Carter 1978; Williams et al. 1995). In addition, we found that specimens from Central America and the western foothills of the Andes in Venezuela, Colombia, and Ecuador (*Tonatia saurophila bakeri*) share similar morphometric characteristics that separate them from specimens from Trinidad and Tobago, Guyana, French Guiana, Brazil and individuals from eastern foothills of the mountain range in Colombia, Ecuador, and Peru (*Tonatia saurophila maresi*), which form morphometrically independent groups (Figs 1, 2). The subspecies *T. saurophila bakeri* presents larger craniodental and external measures than *T. saurophila maresi* (PCA, percentage of variation: 71.3%; DFA, percentage of variation: 86.84%). CCL, GLS, CIL, MTRL, MANDL, and ZB are variables that contribute the most in discriminating these two subspecies (Table 2).

**Table 2.** Measurements (in mm) of *Tonatia bakeri* and *Tonatia maresi*. Measurements are given for the holotypes and for the specimens included in this study (mean and observed range). Holotype data taken from Williams et al. (1995).

Measurements	*Tonatia bakeri*	*Tonatia bakeri*	*Tonatia maresi*	*Tonatia maresi*
	Holotype	specimens	Holotype	specimens
Calcar length	20.00	19.41 (16.37–21.59)	16.00	18.49 (12.10–22.66)
Hindfoot length	15.00	16.22 (13.73–20.00)	13.50	14.69 (11.92–18.00)
Length of ear from notch	14.00	–	30.00	–
Length of forearm	60.60	59.69 (57.45–62.66)	55.00	55.92 (52.70–60.00)
Metacarpal III length	–	50.80 (48.56–53.13)	–	48.11 (43.48–51.73)
Metacarpal IV length	–	51.61 (49.62–54.27)	–	49.21 (44.09–52.36)
Metacarpal V length	–	53.38 (51.43–56.31)	–	50.94 (45.42–54.37)
Tail length	36.00	14.93 (8.38–21.00)	19.00	16.76 (4.55–25.00)
Tibia length	–	29.81 (26.51–31.69)	–	27.00 (24.00–30.77)
Total length	105.00	–	94.00	–
Breadth of braincase	11.70	10.75 (10.46–11.29)	10.50	10.41 (9.68–11.85)
Condylobasal length	25.30	25.51 (24.50–26.73)	24.00	23.79 (22.31–25.17)
Condylocanine length	–	25.03 (24.25–26.00)	–	23.42 (22.28–24.69)
Condyloincisive length	–	25.08 (24.91–26.86)	–	24.22 (22.88–25.55)
Coronoid height	–	7.61 (6.94–8.07)	–	7.07 (6.33–7.71)
Greatest length of skull	30.20	29.73 (28.54–30.78)	28.20	27.90 (26.01–29.38)
Mastoid breadth	13.50	13.07 (12.69–13.45)	12.90	12.55 (11.77–13.35)
Palatal length	–	13.82 (12.59–14.61)	–	12.62 (9.05–13.65)
Palatal width at canines	–	2.47 (1.78–3.13)	–	2.38 (1.65–3.97)
Postorbital constriction breadth	5.60	5.80 (5.41–6.18)	5.30	5.47 (5.05–6.10)
Zygomatic breadth	15.00	13.81 (12.80–14.85)	14.30	13.41 (10.99–14.52)
Breadth across lower incisors	1.60	–	1.80	–
Breadth across upper canines	5.90	5.72 (5.19–6.05)	5.60	5.41 (5.03–5.92)
Breadth across upper molars	9.10	8.56 (8.24–9.16)	8.30	8.12 (7.46–8.88)
Dentary length	–	18.85 (18.29–19.86)	–	17.65 (16.45–18.61)
Height of crown of lower incisor	1.50	–	1.60	–
Length of maxillary toothrow	10.10	10.34 (9.92–10.81)	9.20	9.52 (9.13–10.13)
Mandibular toothrow length	–	11.37 (10.81–11.88)	–	10.51 (9.95–11.11)
Molariform toothrow length	–	7.44 (5.27–8.34)	–	6.44 (4.94–8.06)

### Morphological analyses

**Figure 3.**
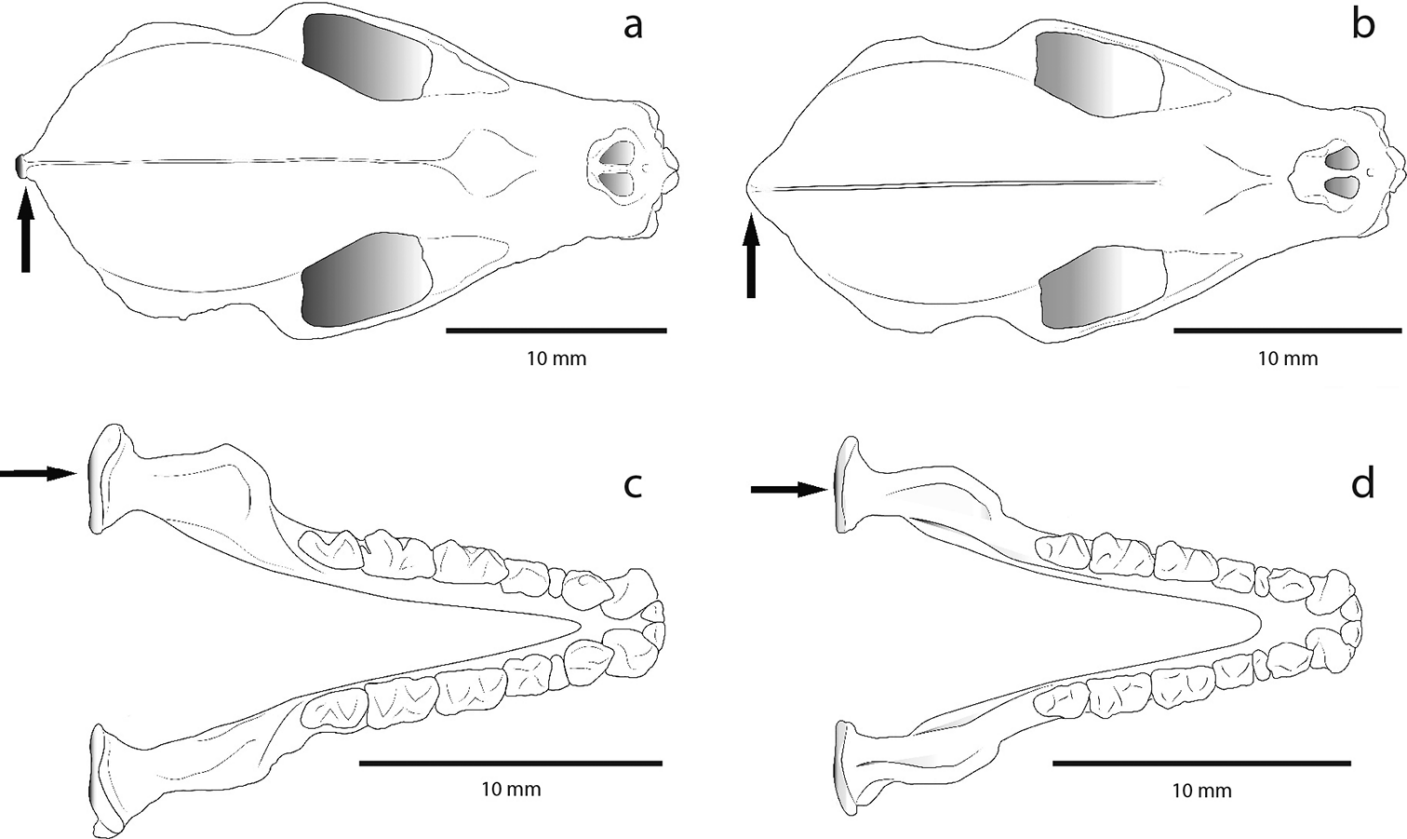
Illustrations showing the differences of the posterior edge of the cranium and the mandibular condyle between *Tonatia bakeri* and *T. maresi*. In the dorsal view of the skull, arrows point to the posterior border of the cranium in **a***T. bakeri* and **b***T. maresi*. In the occlusal view of the mandible, arrows point the mandibular condyle of **c***T. bakeri* and **d***T. maresi*.

**Figure 4.**
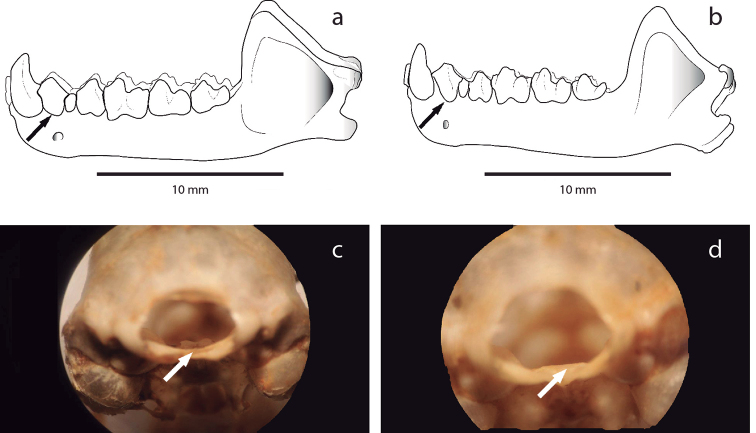
Differences in the separation of the canine with respect to the first premolar, and the development of the clinoid process between *Tonatia bakeri* and *T. maresi*. Illustrations of the lateral view of the mandible, arrows indicate the separation between the canine and the first premolar in **a***T. bakeri* and **b***T. maresi*. In the posterior basioccipital view, arrows indicate the clinoid process in **c***T. bakeri* (QCAZ 9754) and **d***T. maresi* (QCAZ 4972).

Four patterns of cranial and morphological variation were recognized: (1) from a dorsal view of the skull, the posterior edge of the cranial cavity in *T. saurophila maresi* presents a blunt vertex due to presence of a poorly developed sagittal process, whereas *T. saurophila bakeri* presents an acute apex due to presence of a well-developed sagittal process (Fig. 3a, b); (2) in an occlusal view of jaw, the mandibular condyle is feeble in *T. saurophila maresi* and robust in *T. saurophila bakeri* (Fig. 3c, d); (3) in lateral view of mandible, the diastema between canine and first premolar is small in *T. saurophila bakeri*, while in *T. saurophila maresi* the diastema is larger (Fig. 4a, b); and, (4) in view through the foramen magnum (basioccipital view), the clinoid process is well developed in *T. saurophila bakeri* and poorly developed or absent in *T. saurophila maresi* (Fig. 4c, d).

**Figure 5.**
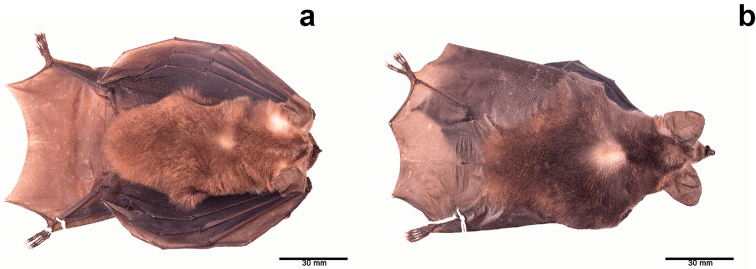
Differences in the dorsal pelage between *Tonatia bakeri* and *T. maresi*. Dorsal view of the body in **a***T. bakeri* (QCAZ 9234) showing lighter and uniform colored pelage, and **b***T. maresi* (QCAZ 15498) showing darker pelage and patches of hair with reddish tips.

Externally, the coloration of the nose leaf, warts of lower lip, and skin surrounding the mouth is lighter in *T. saurophila bakeri*, whereas the skin color in those areas in *T. saurophila maresi* is darker. In addition, *T. saurophila maresi* has darker pelage that present patches of hair with reddish tips, while the pelage in *T. saurophila bakeri* is lighter and uniform in color (Fig. 5).

### Phylogenetic analyses

**Figure 6.**
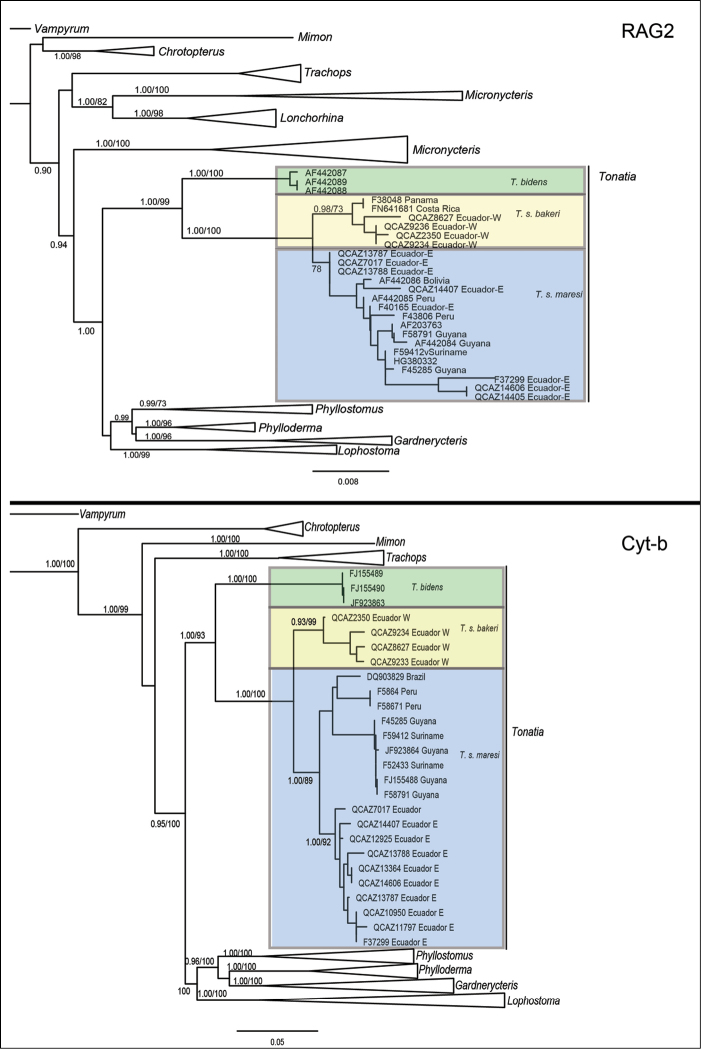
Phylogenetic relationships within *Tonatia* based on Maximum-likelihood derived from the analyses of the RAG2 exon (top) and Cyt-*b* gene (below). Both consensus trees depict three strongly supported clades: *T. bidens*, *T. s. bakeri*, and *T. s. maresi*. The support of each node is represented by the values of posterior probability (PP) and bootstrap (BS), in the phylogeny only the values of PP < 0.90 and BS < 70 are presented. Nodal support is represented by posterior probabilities (<0.90), and bootstrap values (<70).

Maximum Likelihood and BI analyses of the two genes analyzed independently (Cyt-*b* and RAG2; Fig. 6) recovered the genus *Tonatia* as monophyletic and as sister to a clade that included representatives of *Phyllostomus*, *Phylloderma*, *Gardnerycteris*, and *Lophostoma* (Fig. 6). Within *Tonatia*, the monophyly of *T. bidens* was strongly supported. Within *T. saurophila*, two well-supported clades were recovered. One clade included samples from western Ecuador, Panama, and Costa Rica and corresponds to the subspecies *T. saurophila bakeri*. The second clade included samples from eastern Ecuador, Peru, Brazil, and the Guiana Shield and corresponds to *T. saurophila maresi*. Both clades exhibit high support values (Fig. 6).

The Cyt-*b* gene topology showed two clades within *T. saurophila maresi*. Samples from the Guiana Shield, Brazil, and Peru formed a poorly supported clade, while samples from eastern Ecuador formed a clade with high support (Fig. 6). We recovered the sample DQ903829 identified as *T. bidens* (Brazil) nested within the clade of *T. s. maresi*. The exon RAG2 topology for *T. s. maresi* did not record these two clades. The sample FN641681 identified as *P. discolor* (Costa Rica) is located within the clade of *T. s. bakeri* (Fig. 6). In the case of the FN641681 sample, Dávalos et al. (2012) confirmed that the specimen was misidentified. The DQ903829 sample may have the same identification error due to the morphological complexity of the genus *Tonatia* and due to the use of few genetic samples from *T. saurophila* and *T. bidens*. This same misidentification could have occurred in previous works, but, in our case, when using more sequences of *T. saurophila*, the error was notorious.

The average Cyt-*b* pairwise distance between clades from west of the Andes (*T. s. bakeri*) and east of the Andes (*T. s. maresi*) is 7.65% ± 0.65. The clades of *T. s. bakeri* and *T. bidens* exhibit a genetic differentiation of 13.66% ± 1.12, and the clades of *T. s. maresi* and *T. bidens* differ by 13.52% ± 1.02 (Table 3). Levels of intraspecific variation within *T. s. bakeri* and *T. s. maresi* are 1.73% ± 0.29 and 4.01% ± 0.3, respectively (Table 3). Within the *T. s. maresi* clade, the specimens grouped by the country of origin (e.g., Brazil, Ecuador, Peru, and the Guiana Shield) exhibit values between 3.51% and 5.88% (Table 4).

A century has passed since Harold E. Anthony recovered the subfossil material that was used by Koopman and Williams to describe *T. saurophila*. Despite numerous bat surveys since then throughout the West Indies, no additional records of this bat either alive or as subfossil remains have been confirmed. In spite of being known from fragmentary remains, morphological and morphometric differences have been found between the subfossil samples of *T. saurophila* and specimens of *T. bakeri* and *T. maresi*. Based on the aforementioned information we support the recognition of †*Tonatia saurophila* as an extinct full species, with a distribution restricted to Jamaica.

**Table 3.** Corrected genetic distances and intraspecific variation, with standard deviation (bold, in parenthesis), between *Tonatia* species using the Cyt-*b* gene. Values above the diagonal represent the standard deviation.

	***T. maresi***	***T. bidens***	***T. bakeri***
*T. maresi*	**(4.01, 0.37)**	1.02	0.65
*T. bidens*	13.52	**(0.06, 0.06)**	1.12
*T. bakeri*	7.65	13.66	**(1.73, 0.29)**

**Table 4.** Corrected genetic distances between samples of *T. maresi* grouped according to the country of origin. Values above the diagonal represent the standard deviation.

	**Brazil**	**Guiana shield**	**Peru**	**Ecuador**
Brazil		**0.74**	**0.72**	**0.70**
Guiana Shield	4.64		**0.72**	**0.67**
Peru	3.51	5.34		**0.66**
Ecuador	4.84	5.88	5.33	

### Taxonomy

**Family Phyllostomidae Gray, 1825**

**Subfamily Phyllostomidae Gray, 1825**

**Genus *Tonatia* Gray, 1827**

#### † Tonatia saurophila

Taxon classificationAnimaliaChiropteraPhyllostomidae

Koopman & Williams, 1951

17E14D9F-CEBF-5CA7-A3F1-9CBADFDF4A8B

Tonatia saurophila Koopman and Williams 1951: 11.
Tonatia bidens saurophila Koopman 1976: 45.
Tonatia saurophila saurophila Williams, Willig, and Reid 1995: 625.

##### Holotype.

Adult, sex undetermined. Deposited at the American Museum of Natural History (AMNH 147206), collected in 1919–1920 by H. E. Anthony (original field number J ½ T1) in “Wallingford Roadside Cave, Balaclava, St. Elizabeth Parish, Jamaica, British West Indies.” The specimen is a partial mandible from the cave deposits.

##### Paratype.

Adult, sex undetermined. Deposited at the American Museum of Natural History (AMNH 147207), collected in 1919–1920 by H. E. Anthony (original field number J ½ T2) from the same locality as the holotype. A second partial mandible from cave deposits.

##### Additional material.

Adults, sex undetermined. Individuals of undetermined sex, deposited at the American Museum of Natural History (AMNH 147205, 147211, 147212), collected in 1919–1920 by H. E. Anthony from Dairy Cave, Dry Harbour, St. Ann Parish, Jamaica. Fragments of rostra only (no lower jaws). The type series and this material are the only specimens available of *T. saurophila*.

##### Distribution.

**Figure 7.**
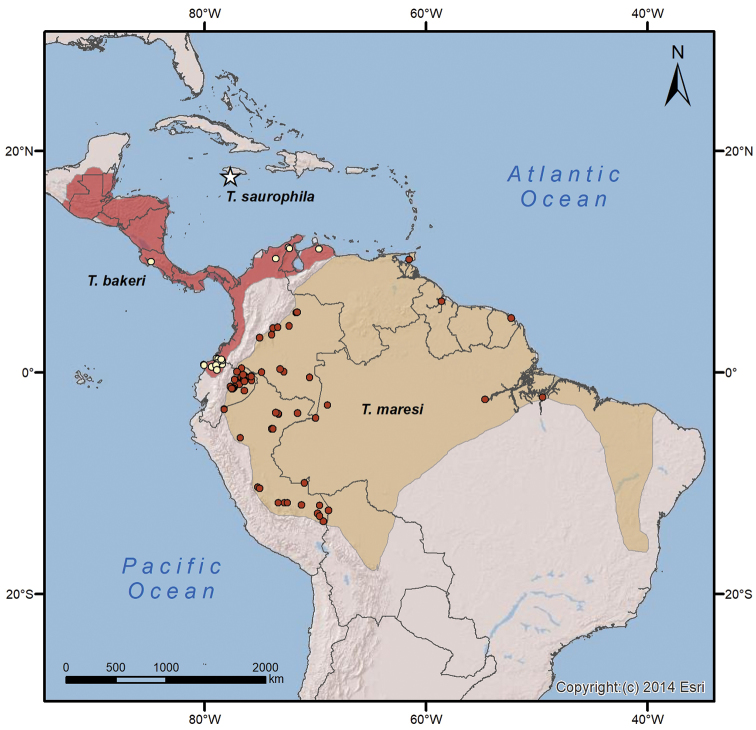
Geographic distribution of *Tonatia saurophila*†, *T. bakeri*, and *T. maresi*. Map of Central and South America showing the geographical distribution of *T. saurophila*† (subfossil records, Jamaica, type locality), *T. bakeri*, and *T. maresi*. Modified from Williams and Genoways (2008), and based on the localities of the specimens included in this study.

The only record of this species is based on the subfossil remains found by H. E. Anthony in the aforementioned caves in Jamaica (Koopman and Williams 1951; Fig. 7).

##### Diagnosis.

Similar to *Tonatia bidens*, but differing in having: the axis of the talonid of m3 straight in an anteroposterior sense, instead of running obliquely in a lingual-labial direction; slightly lower coronoid; more bulbous forehead; a well-developed labial posterior lobe of the last upper premolar; overall size smaller (Koopman and Williams 1951).

##### Description.

The holotype is a partial mandible. The mandible is comprised of the entire dentary bone except for the end of the angular process. All three molars are present along with the last premolar. Complete dental formula of the mandible can be determined from alveoli. There are two small roots, a large canine root and a single small incisor root in front of the last premolar. Also, the coronoid is moderately high. The last premolar is anteriorly squared and therefore the middle premolar is relatively larger (Koopman and Williams 1951). The subfossil fragment of the specimen AMNH 147205 (as shown in the description) includes the entire rostrum, except the extreme anterior end, almost the entire hard palate, and the roots of the teeth except the incisors. Also, the anterior border of the orbit rises obliquely to join the dorsal border. This subfossil also has a slightly bulbous forehead and the presence of an anterior lobe on the last premolar.

Measurements of the holotype taken by Koopman and Williams (1951) are: mandibular toothrow length, 9.8 mm, and depth of ramus behind last molar, 3.1mm. Also, measurements of the paratype taken from Koopman and Williams (1951) are: mandibular toothrow length, 9.5 mm; depth of ramus behind last molar, 2.9 mm; and coronoid height, 6.3 mm.

##### Comparisons.

*Tonatia saurophila* is smaller than any other species within the genus. *Tonatia saurophila* differs from *Tonatia bidens* in having the axis of the talonid of m3 running not obliquely in a lingual-labial direction but straight anteroposteriorly, in having a somewhat more bulbous forehead, and in possessing a well-developed posterior lobe on the last premolar (Koopman and Williams 1951). On the other hand, *T. saurophila* differs from *T. bakeri* and *T. maresi* by having smaller craniodental measurements. Mandibular toothrow length in the holotype of *T. saurophila* is 9.8 mm, while in specimens of *T. bakeri* and *T. maresi*, analyzed in this study, the mandibular toothrow length averaged 11.37 mm and 10.51 mm, respectively. In addition, the coronoid height in the paratype of *T. saurophila* is 6.3 mm, while in specimens of *T. bakeri* and *T. maresi*, analyzed in this study, the coronoid height averaged 7.61 mm and 7.07 mm, respectively. Also, the axis of the talonid of the last molar running not obliquely in a lingual-labial direction, while tends to be oblique in *T. bakeri* and *T. maresi*.

##### Etymology.

The etymology of the term *Tonatia* is unknown (Medellín and Arita 1989). The name *saurophila* is the union of the Latin terms *saurus*, lizard and *philus*, loving. This is because *T. saurophila* was described based on some subfossil material from the “Lizard” stratum of the Wallingford Roadside cave (Williams and Genoways 2008).

##### Remarks.

In 1951, Koopman and Williams considered the fragmentary subfossil material found in the Jamaican caves as a new species. Then, Williams et al. (1995) recognized the Jamaican taxon as the subspecies *Tonatia saurophila saurophila*, along with two other subspecies (*T. saurophila bakeri* and *T. saurophila maresi*). Herein, we recognize *T. bakeri* and *T. maresi* as full species, and support that *T. saurophila* be considered as an extinct monotypic entity, as only subfossil specimens have been recorded in 1920, and there have been no new records since then.

#### Tonatia bakeri

Taxon classificationAnimaliaChiropteraPhyllostomidae

Williams, Willig & Reid, 1995

654527AF-33FC-5FD4-A370-97258F312F55

Tonatia saurophila bakeri Williams et al. 1995: 622.

##### Holotype.

Adult male, deposited at the Museum of Texas Tech University (TTU 39120), collected on 31 January 1983 by R. J. Baker (original field number 1195), 6 km SW of Cana, Darién, Panama, 1200 m. Prepared as skin and skull by M. S. Hafner. Karyotype reference number TK22573. No paratypes were designated by Williams et al. (1995), but several specimens from Mexico, Belize, Costa Rica, Guatemala, Honduras, Nicaragua, Panama, and Venezuela were listed and used in the description.

##### Distribution.

*Tonatia bakeri* is distributed from southeastern Mexico southward into South America to northwestern Ecuador, northern Colombia, and northern Venezuela (west and north of Cordillera de Mérida). The southernmost records of the species are located in the Province of Esmeraldas, Ecuador (Fig. 7).

##### Diagnosis.

*Tonatia bakeri* is distinguished from *T. bidens* and *T. maresi* mainly by craniodental and external measurements. *Tonatia bakeri* is larger than any other species within the genus (Williams et al. 1995; Table 2). The measurements that explain most of the variability are postorbital constriction length, mastoid width, and upper canine width. The skin around the mouth, nose leaf, and warts of the lower lip presents a light coloration. The posterior edge of the cranium presents an acute apex, the mandibular condyles are robust, the diastema between the canine and first lower premolar is absent or not evident, and clinoid process is well developed (Figs 3a, c, 4a, c). Measurements of the holotype taken from Williams et al. (1995), as well as averages of the external craniodental and external measurements of the specimens analyzed in this study, are presented in Table 2.

##### Description.

The holotype has bicolored gray-brown dorsal fur with dark tips; hairs behind ears and neck are bicolored with white bases, and are overall slightly lighter than the rest of the dorsum. Hairs on shoulders are gray-brown, presenting white bases and tips (tricolored). The hairs on the top of head have white tips forming a pale stripe between the ears. Ventral pelage is paler than dorsal pelage; hairs are fawn with lighter tips, but around the throat the fur has a lighter and more uniform color. Dorsal and ventral pelage is dense. Dorsal hairs are longer (12.0 mm) than ventral hairs (5.0 mm). The proximal third of the forearm is covered by short hair (the ventral surface is more densely furred than dorsal), as well as base of the thumbs and proximal side of the feet. The proximal ventral margins of the uropatagium and wing membranes have sparse short hair. The skull of the holotype is complete and in perfect condition, presenting a well-defined sagittal crest.

##### Comparisons.

*Tonatia bakeri* and *T. maresi* differ from *T. bidens* in the presence of a clear line of short fur on the top of the head between ears, a secondary process in the mastoid that partially obscures the base of the bulla (unique within subfamily Phyllostominae), a larger gap between lower canines, a lower crowded appearance of the premolars, and a narrower postorbital constriction. *Tonatia bakeri* differs from *T. maresi* in its narrower breadth across the lower incisors (Williams et al. 1995), presence of an acute apex in the posterior edge of the braincase due to the presence of a well-developed sagittal process, robust mandibular condyles, a reduced or absent space between the canine and the first lower premolar, observable in the mandible body (lateral view), and a well-developed clinoid process (Figs 3a, c, 4a, c). Additionally, *T. bakeri* specimens are larger than those of *T. maresi* (Table 2).

##### Etymology.

The name *bakeri* was coined in recognition to Robert J. Baker for his contributions to the genetics and systematics of the family Phyllostomidae (Williams et al. 1995).

##### Remarks.

Little is known on the natural history of the genus *Tonatia*. It has been reported that it uses hollow trees as day roosts, within which forms monospecific groups, or multispecific groups with other bat species (Williams and Genoways 2008). The diet of *Tonatia* includes arthropods, fruit, and small vertebrates (Tirira 2017).

#### Tonatia maresi

Taxon classificationAnimaliaChiropteraPhyllostomidae

Williams, Willig & Reid, 1995

A1C8791E-AC16-5E5A-AA63-8199F55EC42B

Tonatia saurophila maresi Williams et al. 1995: 623.
Tonatia saurophilla Falcão et al. 2005: 222; incorrect subsequent spelling of T. saurophila Koopman & Williams, 1951.

##### Holotype.

Adult female, deposited at the Museum of Texas Tech University (TTU 9774), collected on 12 July 1969 by R. J. Baker (original field number 318) in Blanchisseuse, Trinidad and Tobago. Prepared as skin, skull, and partial postcranial skeleton by S. L. Williams. No paratypes were designated by Williams et al. (1995), but several specimens from Colombia, Venezuela, Guyana, Suriname, French Guiana, Trinidad and Tobago, Brazil, Peru, and Ecuador were listed and used in the description.

##### Distribution.

*Tonatia maresi* is restricted to South America. It occurs in Venezuela (east and south of Cordillera de Mérida), the Guianas, northeastern Brazil, and along the upper Amazon basin of Colombia, Ecuador, Peru, and Bolivia, as well as in the South American islands of Trinidad and Tobago (Fig. 7).

##### Diagnosis.

*Tonatia maresi* is distinguished from other extant species of *Tonatia* by its smaller craniodental and external measurements. The measurements that explain most of the variability are postorbital constriction length, mastoid width, and upper canine width. The skin around the mouth, nose leaf, and warts of the lower lip present a dark coloration. The posterior edge of the cranium presents a blunt vertex due to the poorly developed sagittal process, mandibular condyles are gracile, the canine and the first lower premolar are separated by a diastema, and the clinoid process is poorly developed or absent (Figs 3b, d, 4b, d). The measurements of the holotype, taken from Williams et al. (1995), as well as the averages of the external craniodental and external measurements of the specimens analyzed in this study are presented in Table 2.

##### Description.

The holotype has dark gray-brown dorsal fur with patches of hair having reddish tips (bicolored). The hairs on the shoulder have white tips and, like the hairs behind the ears and around the base of the neck, present a white base (tricolored). The hairs on the top of head have white tips forming a pale stripe between the ears. Ventral pelage is grayer, and paler, than the dorsal hair and has white tips. The throat region has a uniformly colored hair. The body is densely furred, with the dorsal hairs longer (12.0 mm) than the ventral hairs (5.5 mm). The forearm presents shorter hairs on the proximal half of its length, with the ventral surface being more densely furred. Short, sparse hairs occur on the inner margins of the ventral surfaces of the uropatagium and the wing membranes; short hairs also occur on the thumbs and feet. The skull of holotype is complete, and in perfect condition.

##### Comparisons.

Specimens of *Tonatia maresi* are smaller than those of *T. bakeri* and *T. bidens*. Additionally, *T. maresi* can be distinguished from *T. bakeri* by its wider breadth across the lower incisors (Williams et al. 1995), by the presence of a blunt vertex in the posterior edge of the braincase due to a poorly developed sagittal process, delicate mandibular condyles, the presence of a space between the canine and the first lower molar, observable in the mandible body (lateral view), and the lacking or poorly developed clinoid process (Figs 3b, d, 4b, d). The coloration of the dorsal pelage on *T. maresi* is dark and usually presents patches of hair with reddish tips; while in *T. bakeri* it is light and uniform in color. The warts on the lower lip are darker in *T. maresi* than in *T. bakeri*.

##### Etymology.

The name *maresi* was coined in recognition to Michael A. Mares for his contributions to the systematics, ecology, and zoogeography of South American mammals (Williams et al. 1995).

##### Remarks.

Little is known about its natural history. The diet of *Tonatia* includes various arthropods such as crickets, cicadas, and spiders. Additionally, they consume fruit, and small vertebrates such as lizards and birds (Tirira 2017). The species roosts in hollow trees, forming monospecific groups, or multispecific groups with other bat species (Williams and Genoways 2008). Recently, three species of ectoparasites (*Mastoptera minuta*, *Pseudostrebla greenwelli*, and *Strebla tonatiae*: family Streblidae) were found in specimens of *Tonatia* in the Reserva Natural La Palmita, Department of Casanare, in the Colombian Llanos. This locality occurs within the range of *T. maresi* (Liévano-Romero et al. 2019).

## Discussion and conclusion

Our study shows that, within extant *Tonatia saurophila* populations, there are two clearly differentiated genetic lineages, namely, the lineage of *Tonatia maresi*, which includes samples from eastern Venezuela, Colombia, Ecuador, Peru, Trinidad and Tobago, northeastern Brazil, and the Guiana Shield, and the lineage of *Tonatia bakeri*, which includes samples from Costa Rica, Colombia, Venezuela, and western Ecuador (Fig. 6). These lineages were formerly considered to be subspecies of a single species (*T. saurophila maresi*, *T. saurophila bakeri*; Williams et al. 1995). For decades, the recognition of three subspecies in *Tonatia saurophila* was commonly accepted; however, the integration of morphological, morphometric, and molecular evidence indicates that *T. bakeri* and *T. maresi* are two well-supported and distinguishable taxa. In addition to being dissimilar in size and morphology, they differ genetically at the 7.65% level, a percentage within the 3.3%–14.7% range of genetic distances known to separate sister species of mammals when the Cyt-*b* gene is considered (Baker and Bradley 2006). More recent works that resolve the taxonomic status of species in Phyllostomidae have shown similar genetic differences ranges to those of this study, such as between *Sturnira burtonlimi* and *S. adrianae*: 3.93% + 0.25, *Sturnira hondurensis* and S. *ludovici*: 5.74% + 0.46 (Molinari et al. 2017), and, specifically in the subfamily Phyllostominae, *Lophostoma silvicolum* and *L. evotis*: 5.02% + 0.49; *Lophostoma carrikeri* and *L. brasiliense*: 12.78% + 0.97 (Camacho et al. 2016); *Gardnerycteris crenulatum* and *G. koepckeae*: 11.2% + 1.0 (Hurtado and D’Elia 2018).

According to simultaneous phylogenetic analyses, it is estimated that diversification between bat genera of the subfamily Phyllostominae occurred during beginning and mid-Miocene (23–16.9 Ma; Hoffmann et al. 2008). Specifically the split between *Tonatia* and a clade that included *Artibeus* (Stenodermatinae) and *Anoura* (Glossophaginae) is estimated to have occurred 22 Ma ago (Teeling et al. 2005). At the end of this period, there were probably several ancestral migration processes of *Tonatia* throughout northern South America (as also occurred with *Carollia*), especially in the Northern Andes (Hoffmann and Baker 2003). The divergence between *T. bakeri* and *T. maresi* may have occurred due to a process of allopatric speciation, in which an ancestral population split into two separate lineages as a result of the uplift of the Andes, although this separation had to have happened very recently, along with the rising of the Northern Andes. The Central Andes of Peru and Bolivia and the Northern Andes of Ecuador, Colombia, and Venezuela showed complex landscape transformations during the Miocene and Pliocene. However, the Central Andes experienced its most surface uplift in the late Miocene–Pliocene between 25–14 Ma ago, and the Northern Andes, with a different tectonic history, experienced rapid elevations between 2 and 5 Ma, reaching its modern elevations by around 2.7 Ma (Gregory-Wodzicki 2000).

We speculate that at the beginning of the Pliocene, *Tonatia* may have taken advantage of the newly formed Isthmus of Panama and all subsequent biogeographic processes (i.e. forests expansions) to colonize Central America and some Caribbean islands, including Jamaica (Hoffmann and Baker 2003; Arita et al. 2014; Leigh et al. 2014). Finally, glaciations and tectonic activity in the Andes, during the Pliocene and Early Pleistocene, could have facilitated vicarious speciation within *Tonatia saurophila* (Chesser 2000). However, with the Andes uplift, a reduction in temperature and a shortage of resources at high altitudes possibly were impediments for migratory processes and gene flow to occur between western and eastern populations (Graham 1990). This kind of speciation process has been proposed for other bat species, such as *Artibeus jamaicensis* (Larsen et al. 2007), *Carollia castanea* (Vaca 2016), *Uroderma bilobatum* (Hoffmann et al. 2003), and *Gardnerycteris crenulatum* and *G. keenani* (Hurtado and D’Elia 2018). In the case of *Tonatia maresi*, relationships between the Amazon and the Guiana Shield samples are not clear yet, since the mitochondrial (Cyt-*b*, Fig. 6) and nuclear (RAG2, Fig. 6) gene trees are not congruent. The relatively high level of genetic differentiation may indicate the existence of more than one species.

Despite numerous bat surveys throughout the West Indies in recent years, no new records of *Tonatia saurophila* have been confirmed. *Tonatia* specimens, recorded in Trinidad and Tobago (AMNH 180261–180264 and 182923) have been identified as *T. maresi* on the basis of their morphometric characteristics. Morphometric differences were found between subfossil samples of *T. saurophila* and specimens of *T. bakeri* and *T. maresi* (also considering the individuals collected in Trinidad and Tobago). For example, in *T. saurophila* the mandibular toothrow length and the coronoid height are smaller than in *T. bakeri* and *T. maresi*. These facts support the recognition of *Tonatia saurophila* (formerly classified as *T. saurophila saurophila*) as a full extinct species, with a distribution restricted to Jamaica.

In the Neotropics, more studies on the richness, distribution, and conservation status of the species are urgently needed. Diversity of better-known groups should be studied continuously and consistently, given increasing rates of habitat loss and global climate change. In bats, some of the recently described species were formerly recognized and treated as synonyms or subspecies until extensive mammal collections reviews showed that they were indeed different species (Solari and Martínez 2014). The conservation of biodiversity requires accurate and up to date studies of the taxonomy, distribution, and habitat preferences of species in order to effectively manage and protect them.

### Taxonomic perspectives

With the elevation of *T. bakeri* and *T. maresi* to the species category, the genus *Tonatia* now includes three extant species, including *T. bidens*, and one extinct species.

## Supplementary Material

XML Treatment for Tonatia saurophila

XML Treatment for Tonatia bakeri

XML Treatment for Tonatia maresi

## Appendix 1

Examined specimens

***Tonatia bakeri***

COLOMBIA – **Cesar** 1 ♂; Valledupar, “Corregimiento Río Seco, Las Palomas”; 10.278N, 73.574W; 276 m a.s.l.; 30 Apr. 2011; ICN 21276. – **Guajira** • 1 ♀; Albania, “Cerrejón, compensation area - Mushaisa sector”; 11.277N, 72.412W; 13 Dec. 2005; ICN 19454 • 1 ♂; Albania, “Cerrejón, Palomino”; 11.177N, 72.304W; 24 Mar. 2006; ICN 19455.

COSTA RICA • 3 ♀♀; Puntarenas, Puntarenas, Palmar; 9.9763N, 84.838W; 2 Feb. 1941; AMNH 139437 / 139438 / 139445 • 2 ♂♂; same data as for preceding; AMNH 139439 / 139440 • 2 ♀♀; same data as for preceding; 1 Feb. 1941; AMNH 139443 / 139444.

ECUADOR – **Carchi** • 1; Tulcán, “Tobar Donoso, vía Anrá”; 1.1892N, 78.488W; 258 m a.s.l.; 22 Sep. 2009; MECN 2948 • 1 ♀; Tulcán, “Tobar Donoso, vía Helipuerto”; 1.1892N, 78.488W; 305 m a.s.l.; 22 Aug. 2010; MECN 3305. – **Esmeraldas** • 1 ♂, 1 ♀; Muisne, Quingue; 0.7231N, 80.078W; 6 Sep. 2005; QCAZM 7423 / 8282 • 1 ♂; San Lorenzo, Comuna San Francisco de Bogotá; 1.2536N, 78.9W; 63 m a.s.l.; 12 Aug. 2004; QCAZM 9233 • 1 ♂; San Lorenzo, San Francisco Santa Rita; 1.2527N, 78.909W; 88 m a.s.l.; 12 Aug. 2004; QCAZM 9235 • 1 ♀; same data as for preceding; 1.0421N, 78.424W; 88 m a.s.l.; 12 Aug. 2004; QCAZM 9236 • 1 ♀, 1 ♂, San Lorenzo, San Lorenzo; 1.2333N, 78.76W; 12 Aug. 2004; QCAZM 9754 / 9755 • 2 ♂♂; Eloy Alfaro, “Playa de Oro, Culo de Negra”; 0.8712N, 78.792W; 113 m a.s.l.; 25 Aug. 2017; QCAZM 17041 / 17044 • 1 ♀; same data as for preceding; QCAZM 17045 • 1 ♀; Eloy Alfaro, “Luis Vargas Torres, Playa de Oro “; 0.8758N, 78.794W; 75 m a.s.l.; 25 Aug. 2017; QCAZM 17042 • 1 ♀; Muisne, Bunche Estero el Aguacate; 0.6496N, 80.033W; 37m a.s.l.; 1 Jun. 2000; MECN 2049 • 1 ♂; Quinindé, Valle del Sade; 0.5177N, 79.341W; 250 m a.s.l.; 10 Mar. 1985; EPN 2038 • 2 ♂♂; same data as for preceding; 13 Mar. 1985; EPN 2039 / 2040 • 1 ♀; Salto del Río Bravo; 0.6813N, 78.946W; 198 m a.s.l.; 10 Aug 1985; EPN 2041. – **Imbabura** • 1 ♂; Ibarra, Verde River; 0.7497N, 78.401W; 750 m a.s.l.; 15 Oct 2000; QCAZM 3810 • 1 ♂; Cotacachi, Cielo Verde; 0.2241N, 78.899W; 630 m a.s.l.; 13 Apr. 2010; EPN 11725.

VENEZUELA • 1 ♂; Falcón, Bolivar, Tocuyo River; 11.121N, 69.683W; 26 Mar. 1938; AMNH 130704.

***Tonatia maresi***

BRAZIL • 1 ♂; Para, Santarem, Tapajos River; 2.4394S, 54.698W; 7 Aug. 1931; AMNH 95429 • 1 ♀; Para, Cametá, “Tocantins River, Ilha do Taiuna”; 2.2540S, 49.512W; 2 Nov. 1931; AMNH 96964 • 3 ♀♀; same data as for preceding; 12 Nov. 1931; AMNH 96997 to 96999 • 1 ♀; same data as for preceding; 13 Nov. 1931; AMNH 97226.

COLOMBIA – **Amazonas** • 1 ♀; Leticia,Caño Boliviano; 4.1005S, 69.933W; 150 m a.s.l.; 23 Jun. 2005; ICN 17874 • 1 ♀; Leticia, “Tarapacá, Hitoma community”; 4.1005S, 69.933W; 84 m a.s.l.; 26 Apr. 2002; ICN 21033 • 1 ♂; same data as for preceding; 29 Apr. 2002; ICN 21034. – **Caquetá** • 1 ♂; Solano,”Mesay River, Puerto Abeja (trocha piscina), Serrania de Chiribiquete”; 0.0753N, 72.806W; 240 m a.s.l.; 2 Feb. 1995; ICN 14619 • 1 ♂; Solano,”Mesay River, Puerto Abeja, (Tepuy), Serrania de Chiribiquete”; 0.3216N, 73.167W; 340 m a.s.l.;30 Jan. 1995; ICN 14623 • 1 ♂; Solano,”Mesay River, Bombonal (rastrojo)”; 0.321631N, 73.167614W; 240 m a.s.l.; 1 Jun. 1994; ICN 14685. – **Casanare** • 1 ♀; Trinidad, “Vereda La Cañada, La Palmita”; 5.4186N, 71.655W; 153 m a.s.l.; 12 Sep. 2008; ICN 19338 • 1 ♂; same data as for preceding; 165 m a.s.l.; 17 Sep. 2008; ICN 19339 • 1 ♀; San Luis de Palenque, “Vereda Guaracura, La Bretaña”; 5.3801N, 71.727W; 125 m a.s.l.; 5 Mar. 2012; ICN 22944 • 1 ♀; Trinidad, “Vereda La Cañada, R. N. La Palmita, caño Guajivo, sendero Morroco”; 5.4186N, 71.655W; 168 m a.s.l.; 25 Jun. 2014; ICN 23103. – **Huila** • 1 ♂; Baraya, “El Cruce, Las Delicias”; 3.1547N, 75.026W; 750 m a.s.l.; 8 Apr. 1994; ICN 13625. – **Meta** • 1 ♀; Acacías, “Vereda San José, Colegio Departamental Agropecuario”; 4.0062N, 73.797W; 625 m a.s.l.; 8 Dec. 1985; ICN 9692 • 1 ♂; San Juan de Arama, “Serranía de La Macarena, caño La Curia “; 3.4003N, 73.941W; 500 m a.s.l.; 27 Apr. 1988; ICN 10251 • 1 ♀; same data as for preceding; 22 May. 1988; ICN 10252 • 1 ♀; same data as for preceding; 1 May. 1992; ICN 12045 • 1 ♀; same data as for preceding; 9 Aug. 1988; ICN 12054 • 1 ♂; same data as for preceding; 11 Jul. 1988; ICN 12056 • 1 ♂; Puerto Gaitán, Carimagua Investigation Centre; 4.1886N, 72.357W; 150 m a.s.l.; 1 Sep. 2005; ICN 18425 • 1 ♀; Villavicencio, “Barrio El Manantial, wetland Reserva Parque El Coroncero”; 4.0740N, 73.367W; 285 m a.s.l.; 9 May 2003; ICN 18456. – **Putumayo** • 1 ♂; Puerto Leguízamo, “Vda. El Triunfo, km 19 La Taguita, La Distancia (Pablo Yara)”; 0.0394N, 74.830W; 203 m a.s.l.; 27 May. 2015; ICN 22007. – **Vaupes** • 1 ♀; La Libertad, Serranía de Taraira; 0.4264S, 70.496W; 310 m a.s.l.; 8 Aug. 1993; ICN 12731.

ECUADOR – **Morona Santiago** • 1 ♂; San Juan Bosco, “Kunkuki community, 25 min to the mountain”; 3.3213S, 78.211W; 1186 m a.s.l.; 2 Jan 2018; QCAZM 17373 • 1 ♀; same data as for preceding; 3.3277S, 78.212W; 1216 m a.s.l.; 3 Jan 2018; QCAZM 17374. – **Orellana** • 1 ♂; Yasuní National Park; 0.5629S, 76.463W; 220 m a.s.l.; 10 Mar. 2001; QCAZM 4972 • 1 ♂; same data as for preceding; 5 Mar. 2001; QCAZM 7569 • 1 ♂; same data as for preceding; 0.38S, 76.3W; 220 m a.s.l.; 5 Mar 2001; QCAZM 7574 • 1; same data as for preceding; 0.6768S, 76.936W; 246 m a.s.l.; 3 Jun. 2001; QCAZM 12433 • 1 ♂; same data as for preceding; 0.6722S, 76.399W; 217 m a.s.l.; 31 May 2013; QCAZM 14028 • 1 ♀; Orellana, Dumbique; 0.5181S, 76.122W; 217 m a.s.l.; 18 Jan 2009; QCAZM 10950 • 1 ♂; Loreto, Loreto; 0.6803S, 77.199W; 316 m a.s.l.; 20 Dec. 2009; QCAZM 11797 • 1 ♂; Joya de los Sachas, Comuna San Antonio; 0.3939S, 76.66W; 279 m a.s.l.; 8 Mar. 2013; QCAZM 13787 • 1 ♀; Orellana, Pre. Coop. Cononaco; 1.0013S, 76.973W; 279 m a.s.l.; 24 Mar. 2013; QCAZM 13788 • 1 ♂; Orellana, Dayuma Shiripuno River; 1.0598S, 76.867W; 246 m a.s.l.; 1 Jul. 2013; QCAZM 14407 • 1 ♀; Francisco de Orellana, “Yasuní National Park, 37 km S Pompeya South”; 0.6333S, 76.466W; 11 May. 1995; MECN 662 • 1 ♂; Francisco de Orellana, “Yasuní National Park, 42 km S, 1 km E Pompeya South”; 0.6833S, 76.433W; 12 Oct. 1995; MECN 762 • 1 ♂; Aguarico, “Yasuní National Park, 66 km S Pompeya South”; 0.8S, 76.4W; 8 Jun. 1996; MECN 976 • 1 ♀; Aguarico, Garzacocha Yasuní National Park; 0.7283S, 75.752W; 27 Sep. 1988; EPN 3833 • 1 ♂; Loreto, Caimitoyaku; 0.6137S, 77.251W; 510 m a.s.l.; 12 Sep. 1996; EPN 5113. – **Pastaza** • 1 ♂; Arajuno, Operation Area AGIP - Block 10; 1.4531S, 77.441W; 380 m a.s.l.; 27 Mar. 2012; QCAZM 12843 • 2 ♂♂; Arajuno, Operation Area AGIP - Block 10; 1.506S, 77.509W; 350 m a.s.l.; 16 May. 2012; QCAZM 12925 / 12930 • 1 ♀; Arajuno, Tarangaro; 1.4006S, 77.383W; 23 Jun. 2012; QCAZM 12975 •1 ♀; Arajuno, Oglán, 1.2569S, 77.654W; 18 Oct. 2012; QCAZM 13364 • 1 ♀; Pastaza, Lorocachi; 1.666S, 76.418W; 223 m a.s.l.; 31 May. 2013; QCAZM 14388 • 1 ♂; Arajuno, AGIP Oil installations. Block 10; 1.27S, 77.26W; 393 m a.s.l.; 1 Feb. 2013; QCAZM 14605 • 1 ♀; Arajuno, Tarangaro; 1.4006S, 77.383W; 320 m a.s.l.; 27 Nov 2013; QCAZM 14606 • 2 ♂♂; Arajuno, Operation Area AGIP - Block 10; 1.4636S, 77.45W; 370 m a.s.l.; 2 Apr. 2015; QCAZM 15498 / 15499 • 1 ♂; same data as for preceding; 6 Apr. 2015; QCAZM 15500 • 1 ♂; Arajuno, Operation Area AGIP - Block 10; 1.4748S, 77.531W; 455 m a.s.l.; 20 Jul. 2015; QCAZM 15793 • 1 ♂; same data as for preceding; 459 m a.s.l.; 23 Jul. 2015; QCAZM 15794. – **Sucumbios** • 1 ♂; Cuyabeno, Cuyabeno River Bridge; 0.32S, 76.32W; 230 m a.s.l.; 22. Jan 2004; QCAZM 6853 • 1 ♀; same data as for preceding; 24 Jan. 2004; QCAZM 6875 • 1 ♂; same data as for preceding; 25 Jan. 2004; QCAZM 6938 • 1 ♂; Cuyabeno, Zábalo; 0.3181S, 75.766W; 233 m a.s.l.; 17 Mar. 2004; QCAZM 7017 • 1 ♀; Cuyabeno, Coop. Tierras Orientales; 0.3129S, 76.366W; 233 m a.s.l.; 8 Nov. 2008; QCAZM 10831 • 1 ♀; Shushufindi, Limoncocha - Biological Reserve; 0.4N, 76.633333W; 28 Sep. 1978; EPN 3425; • 1 ♂; Cuyabeno, “Cofán Zábalo Community, Aguarico River”; 0.3666S, 75.768W; 235m a.s.l.; 13 Oct. 1991; EPN 4481; • 1 ♀; Lago Agrio,”Aguarico River, Pozo Zafiro”; 0.0574N, 77.081W; 17 Mar. 1996; EPN 5008 • 1 ♀; Shushufindi, Panacocha Pañayaku River BPP; 0.203S, 76.526W; 229m a.s.l.; 21 Feb. 2010; EPN 11681.

FRENCH GUIANA • 1♀; Cayenne, Sinnamary, Paracou; 4.9333N, 52.333W; 7 m a.s.l.; 9 Jul. 1991; AMNH 266047 • 1 ♀; same data as for preceding; 31. Aug. 1993; AMNH 267429 • 1 ♂; same data as for preceding; 10 Sep. 1993; AMNH 267431 • 1 ♂; same data as for preceding; 24 Oct. 1994; AMNH 267909 • 1 ♂; same data as for preceding; 3 Oct. 1994; AMNH 267912 • 1 ♀; same data as for preceding; 7 Oct. 1994; AMNH 267914.

GUYANA • 1 ♂; Cuyuni Mazaruni Region, Bartica, Kartabo Point; 6.4N, 58.616W; 30 Aug. 1922; AMNH 64164.

PERU – **Cusco** • 1 ♂; La Convención, “Camisea, Pagoreni”; 11.779S, 72.783W; 450 m a.s.l.; 9 Apr. 1998; MUSM 13970 • 1 ♀; La Convención, 2 km southwest of C.N. Tangoshiari; 11.779S, 73.341W; 1000 m a.s.l.; 14 May 1998; MUSM 13399 • 1 ♀; La Convención, “Camisea, San Martín”; 11.772S, 72.522W; 475 m a.s.l.; 12 May. 1997; MUSM 13971. – **Loreto** • 1 ♀; Requena, “Lagarto, Ucayali River”; 5.07S, 73.91W; 17 Jan. 1928; AMNH 76558 • 1 ♀; same data as for preceding; 20 Jan. 1928; AMNH 76559 • 1 ♀; same data as for preceding; 20 Jan. 1928; AMNH 76560 • 1 ♂; same data as for preceding; 24 Jan. 2012; AMNH 278467 • 1 ♂; same data as for preceding; 28 Jan. 2012; AMNH 278484 • 1 ♀; same data as for preceding; CEBIO 110 • 1 ♂; Balsapuerto, “Cerro Escalera, Cachiyacu Camp”; 5.9014S, 76.777W; 2520 m a.s.l.; 25 Sep. 2013; FMNH 233361 • 1 ♀; Requena, Jenaro Herrerra; 5.0806S, 73.819W; MUSM 5549 • 1 ♂; Maynas, Lagartococha River Catalino camp; 3.7368S; 73.312W; 26 Feb. 1994; MUSM 21293 • 1 ♂; Maynas, Lagartococha River Catalino camp; 3.6541S, 71.621W; 2 Mar. 1994; MUSM 21294 • 1 ♀; Maynas, 20 km south of Diamante Azul town; 3.7066S, 73.345W; 139 m a.s.l.; 15 Oct. 2008; MUSM 26538 • 1 ♀; Maynas, 13 km northwest of Albarenga town; 3.6315S, 73.591W; 145 m a.s.l.; 20 Jan. 2009; MUSM 26578 – **Madre de Dios** • 2 ♂♂; Manu, Pakitza; 11.952S, 71.249W; 350 m a.s.l.; 6 Nov. 1990; MUSM 6811 / 6812 • 1 ♀; Manu,Pakitza; 11.952S, 71.249W; MUSM 6815 • 1 ♀; Tambopata, Santuario Nacional Pampas del Heathi; 11.997S, 69.597W; 200 m a.s.l.; 9 Jun. 1996; MUSM 11715 • 1 ♀; Tambopata, Santuario Nacional Pampas del Heathi; 2.9544S, 68.9W; 200 m a.s.l.; 9 Jun. 1996; MUSM 11717 • 1 ♀; Manu, “Santuario Nacional Pampas del Heath, Picoplacha”; 12.439S, 68.806W; 6 Jun. 1992; MUSM 12860 • 1 ♂; Manu, “CICRA, Cruce Sachavaca, Bajío”; 12.721S, 69.786W; 199 m a.s.l.; 25 Oct. 2005; MUSM 26094 • 1 ♀; Manu, Trocha Trompeteros; 12.948S, 69.606W; 148m a.s.l.; 22 Apr. 2006; MUSM 26133. – **Pasco** • 1 ♂; Oxapampa, Iscozacín; 10.348S, 75.193W; 15 Jul. 1988; MUSM 450 • 2 ♀; Oxapampa, “Cerro Chontilla, 5 km west Shiringamazu, road to Iscozacin”; 10.484S, 75.043W; 930 m a.s.l.; 6 Jul. 1992; MUSM 10345 • 1 ♂; same data as for preceding; 4 Jul. 1992; MUSM 10346. – **Puno** • 1 ♀; Carabaya, “Coasa, Boca de Qa. Ursulinda del Río Candamo”; 13.422S, 69.28W; 400 m a.s.l.; 10 Apr. 1999; MUSM 15829. – **Ucayali** • 1 ♀; Purús, “Curanja River, Balta”; 9.9666S, 70.966W; 300 m a.s.l.; 3 Apr. 1971; MUSM 1189.

TRINIDAD AND TOBAGO • 1 ♂, 3 ♀; Saint Patrick, “Trinidad Island, Siparia”; 10.183N, 61.55W; 13 Jan. 1959; AMNH 180261 to 180264 • 1 ♀; Saint Patrick, “Trinidad Island, La Brea”; 11.55N, 60.183W; 7 May. 1959; AMNH 182923.
